# Predictive Indicators of Survival in Patients With Surgically Resected Lung Carcinoid Tumors at a Greek Medical Center

**DOI:** 10.7759/cureus.10300

**Published:** 2020-09-07

**Authors:** Vasiliki E Georgakopoulou, Eleftherios Zygouris, Christos Nikokiris, Christos Damaskos, Aikaterini Pierrakou, Nikolaos Garmpis, Anna Garmpi, Pagona Sklapani, Aikaterini Aravantinou, Nikolaos Trakas, Jim Janinis, Jubrail Dahabreh

**Affiliations:** 1 Pulmonology Department, Laiko General Hospital, Athens, GRC; 2 1st Pulmonology Department, Sismanogleio Hospital, Athens, GRC; 3 Oncology, Athens Medical Center, Athens, GRC; 4 Intensive Care Unit, Sismanogleio Hospital, Athens, GRC; 5 Renal Transplantation Unit, Laiko General Hospital, N.S. Christeas Laboratory of Experimental Surgery and Surgical Research, Medical School, National and Kapodistrian University of Athens, Athens, GRC; 6 Pathology, Athens Medical Center, Athens, GRC; 7 Second Department of Propedeutic Surgery, Laiko General Hospital, Medical School, National and Kapodistrian University of Athens, Athens, GRC; 8 First Department of Propedeutic Internal Medicine, Laiko General Hospital, Medical School, National and Kapodistrian University of Athens, Athens, GRC; 9 Department of Cytology, Mitera-Hygeia Hospital, Athens, GRC; 10 Internal Medicine Department, Laiko General Hospital, Athens, GRC; 11 Biochemistry Department, Sismanogleio Hospital, Athens, GRC; 12 Thoracic Surgery, Athens Medical Center, Athens, GRC

**Keywords:** pulmonary carcinoids, ki67, type of surgery

## Abstract

Introduction

Lung carcinoid tumors are neuroendocrine neoplasms, less frequent than other lung tumors. They are subdivided into typical carcinoids (TC) and atypical carcinoids (AC), according to the rate of mitosis and the presence of necrosis. Lung carcinoids are often asymptomatic and only discovered incidentally. They may also present with cough, wheezing, asthma, and chronic obstructive pulmonary disease, chest pain, and hemoptysis depending on the location of the tumor and, less commonly, present with carcinoid syndrome. In our study, we describe the clinical and pathological features of patients with surgically resected lung carcinoids at our institution over a period of 14 years. We also examine if these features, including age, gender, tumor size, type of carcinoid, stage, nodal involvement, and Ki-67 expression are associated with patients' survival.

Materials and methods

We retrospectively reviewed patients that underwent surgery with a final histologic diagnosis of a pulmonary carcinoid tumor from March 2005 to March 2019. The evaluation included history, physical examination, chest radiographs, computerized tomography of the chest, upper abdomen, and brain, and bone scintiscan. All specimens resected during the surgical procedures were sent for pathological examination, including mediastinal and hilar lymph nodes. The patients' age, gender, tumor size, type of carcinoid, nodal involvement, stage, and Ki-67 expression were recorded and correlated to the patients' survival rates.

Results

The study included 108 patients - 52 males and 56 females - with a mean age of 51.5 years (range 11-80 years). Atypical carcinoid was the diagnosis in 28 patients (16 males and 12 females) and 80 patients had the diagnosis of typical carcinoid (36 males and 44 females). Tumor size was ≤3.7 cm in 84 patients (68 with TC and 16 with AC) and >3.7 cm in 22 patients (12 with TC and 10 with AC). Sixteen patients had nodal deposits, 12 in N1 nodes and four in N2 nodes. Eighty patients were classified in stage I, 18 patients in stage II, and 10 patients in stage III. None of the patients had distant metastases. The Ki-67 proliferation index was examined in 84 specimens and Ki-67 was <2.5 in 50 patients and ≥2.5 in 34 patients. Of the 108 patients, eight died, all with disease-related death. According to the Cox regression univariate analysis, four factors were correlated to shorter survival: atypical histology, tumor size >3.7 cm, nodal involvement, and advanced stage

Conclusions

In conclusion, we found that histological type, tumor size, nodal involvement, and stage are associated with survival in patients with surgically resected lung carcinoids without distant metastases. Other parameters, such as age at operation, gender, and Ki-67 index, did not have a role in survival in these patients according to the Cox regression univariate analysis.

## Introduction

Lung carcinoid tumors are rare neoplasms, accounting for 1%-2% of all lung tumors that are lung neuroendocrine tumors. They are subdivided into typical carcinoids (TC) and atypical carcinoids (AC), according to the rate of mitosis and the presence of necrosis [1]. Typical carcinoids are characterized by carcinoid morphology, less than two mitoses per 2 mm^2^ (10 high-power fields), and lack of necrosis. Atypical carcinoids are tumors with a carcinoid morphology, two to 10 mitosis per 2 mm^2^ (10 high-power fields), and/or necrosis [2].

The majority of pulmonary carcinoids (80%) occur centrally and all pulmonary carcinoids are malignant and can give distant metastases. TC usually metastasize to the liver and bone and AC metastasize to the bone, brain, liver, soft tissue, adrenal, and spleen [3]. The mechanisms of carcinoid tumor development are not clearly defined, but there is evidence that some cases develop in the setting of proliferating pulmonary neuroendocrine cells through diffuse idiopathic pulmonary neuroendocrine cell hyperplasia (DIPNECH) and tumorlets. A family history of carcinoid tumors and carrying the Multiple Endocrine Neoplasia type 1 (MEN1) gene are risk factors for carcinoid development. Pulmonary carcinoids can occur at any age, accounting for the majority of pulmonary tumors in childhood, with a mean age of 50 years at diagnosis and equal distribution between males and females [3].

Lung carcinoids are often asymptomatic and only discovered incidentally. They may also present with cough, wheezing, asthma, and chronic obstructive pulmonary disease, chest pain, and hemoptysis depending on the location of the tumor and less commonly present with carcinoid syndrome. In rare cases, they can present with Cushing syndrome and acromegaly due to the overproduction of adrenocorticotropic hormone (ACTH) and growth hormone-releasing hormone (GHRH), respectively [4].

The diagnosis of lung carcinoids is based on bronchoscopy with a fiberoptic bronchoscope, radiological assessment, and biochemical evaluation. Bronchoscopy plays a key role in the diagnosis of carcinoids, as in most cases, the tumor is centrally located and visible at endoscopy, allowing bronchoscopic biopsy [5]. Τhe gold standard for the radiological detection of lung carcinoids is a computed tomography (CT) scan, in which when bronchial involvement is present, secondary findings can be atelectasis, bronchiectasis, and hyperlucency [6].

Fluorodeoxyglucose (FDG) positron emission tomography (PET) may be helpful in distinguishing carcinoids from high-grade neuroendocrine tumors such as small cell or large cell neuroendocrine tumors [7]. Octreotide single-photon emission CT and other imaging techniques, such as gallium-labeled somatostatin analogs, are useful in the detection of lung carcinoids [8], and imaging for somatostatin receptors using Indium-111-labeled-octreotide may increase the sensitivity for the diagnosis, staging, and follow-up of lung carcinoids [9]. In addition, specific tests are performed when symptoms suggest hormonal secretion, including urinary dU-5-hydroxy indol-acetic acid in patients with carcinoid syndrome, serum cortisol, 24-hour urine-free cortisol, GHRH, and insulin growth factor (IGF)-I when signs of acromegaly are present and ACTH levels in patients with Cushing syndrome [10].

Staging of pulmonary carcinoids is performed according to the Union Internationale Contre le Cancer/American Joint Committee on Cancer (UICC/AJCC) tumor, node, metastasis (TNM) system [10]. The treatment of choice for patients with lung carcinoids is surgical resection [10]. Lobectomy is the surgical procedure of choice. Bronchoplastic techniques are usually required for carcinoids arising from main bronchi or lobar bronchi while a wedge resection is performed in peripheral carcinoids [4].

The National Comprehensive Cancer Network guidelines recommend adjuvant chemotherapy with or without radiation in stage III atypical carcinoids while the European Neuroendocrine Tumor Society suggests adjuvant treatment only in atypical carcinoids with positive lymph nodes [4].

Multiple parameters have been reported as prognostic factors for the survival of lung carcinoid tumors. These parameters include metastases, tumor size, histologic subtype, age, mediastinal lymph node status, type of surgical procedure, and Ki-67 expression. The Ki-67 antigen is the product of the MKI67 gene related to cell proliferation, which has a value in discriminating pulmonary carcinoids from high-grade neuroendocrine tumors in small biopsies [11].

In our study, we describe the clinical and pathological features of patients with surgically resected lung carcinoids at our institution over a period of 14 years. We also examine if these features, including age, gender, tumor size, type of carcinoid, stage, nodal involvement, and Ki-67 expression are associated with the patients' survival.

## Materials and methods

We retrospectively reviewed patients that underwent surgery with a final histologic diagnosis of a pulmonary carcinoid tumor from March 2005 to March 2019. The evaluation included history, physical examination, chest radiographs, CT of the chest, upper abdomen, and brain, and bone scintiscan. All patients had a preoperative evaluation with a fiberoptic bronchoscope, and in some patient's endoscopic biopsy was performed while fine-needle aspiration biopsy was performed in peripheral tumors. All specimens resected during the surgical procedure were sent for pathological examination, including mediastinal and hilar lymph nodes. Tumors were classified into typical and atypical carcinoids according to the World Health Organization (WHO) classification of tumors of the lung, pleural, thymus, and heart, 2015 [12]. The tumor stage was determined according to the 8th ΤΝΜ staging system of lung cancer [13]. Immunohistochemical staining against Ki67 was performed in the clinical pathology laboratory (Department of Pathology, Athens Medical Group). Briefly, hematoxylin- and eosin-stained sections were prepared from the paraffin-embedded tissue blocks to identify the diagnostic area. Formalin-fixed paraffin sections that were 2 µm thick were used for IHC staining. Stainings of slides were performed in the AutoStainer Link 48 DAKO instrument (Agilent Technologies, Santa Clara, CA). The sections were deparaffinized, and antigen retrieval was performed using the Envision Flex Target Retrieval Solution High pH (Agilent Technologies). The primary antibodies were incubated, and the immunoreactions were detected using EnVision Detection Systems (Dako, Agilent Pathology Solutions). All slides were counterstained with Mayer’s hematoxylin (Dako). The patients' age, gender, tumor size, type of carcinoid, nodal involvement, stage, and Ki-67 expression were recorded and correlated to the patients' survival rates. For statistical analysis, the Statistical Package for Social Sciences software (SPSS for Windows, version 13.0, SPSS Inc, Chicago, III) was used. Life tables were estimated by Kaplan-Meier statistics, and survival curves were compared using the log-rank test. Survival was measured in units of months from surgery. The Cox hazard-regression model, including relative risk, probability, and 95% confidence interval, was used for univariate analysis for the prognostic factors. All p-values are two-sided, and 5% was chosen as the level of statistical significance.

## Results

The study included 108 patients - 52 males and 56 females - with a mean age of 51.5 years (range 11-80 years). Atypical carcinoid was the diagnosis in 28 patients (16 males and 12 females) and 80 patients had the diagnosis of typical carcinoid (36 males and 44 females). The tumor was in the left lung in 38 (35.1%) patients, in the right lung in 68 (63%) patients, and in the trachea in two (1.9%) patients. The surgical procedures included 72 lobectomies, 10 pneumonectomies, 12 bronchoplasties, two pleuropneumonectomies, four bilobectomies, two tumors resections, two wedge resections, and four bronchus resections. None of the patients received neoadjuvant or adjuvant therapy or radiation therapy. Tumor size was determined in 106 patients on initial diagnosis. Tumor size was ≤3.7 cm in 84 patients (68 with TC and 16 with AC) and >3.7 cm in 22 patients (12 with TC and 10 with AC). Nodal involvement was examined in 104 patients. Sixteen patients had nodal deposits, 12 in N1 nodes, and four in N2 nodes. Eighty patients were classified in stage I, 18 patients in stage II, and 10 patients in stage III. None of the patients had distant metastases. The Ki-67 proliferation index was examined only in 84 specimens because the rest cases were older and the reagents were not available. Ki-67 was <2.5 in 50 patients and ≥2.5 in 34 patients (Table 1).

**Table 1:** Demographic and histopathological characteristics of patients AC: atypical carcinoid; TC: typical carcinoid

Variable	TC	AC	ALL
Gender			
Male	36 (33.3%)	16 (14.8%)	52 (48.1%)
Female	44 (40.7%)	12 (11.1%)	56 (51.9%)
Age			
Mean	47.5 (76)	56.1 (24)	51.5 (108)
Median	50	65	52
Range	Nov-80	15-80	Nov-80
Location			
Left	28 (25.5%)	10 (9.1%)	38 (35.1%)
Upper lobe	12	6	18
Lower lobe	12	4	16
Whole lung	4	0	4
Right	50 (45.5%)	16 (14.5%)	68 (63%)
Upper lobe	10	4	14
Lower lobe	12	2	14
Middle lobe	16	2	18
Whole lung	0	8	8

**Figure 1:**
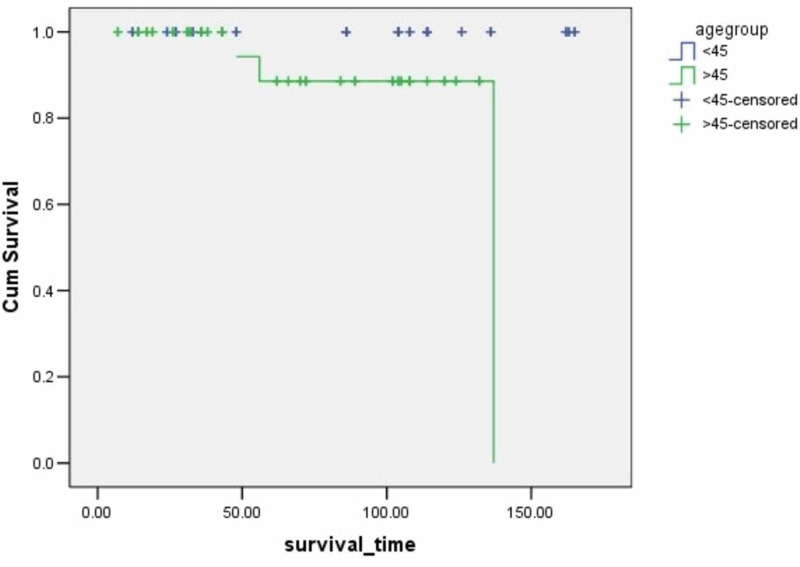
Survival rates based on age The x-axis indicates months from surgery and the y-axis indicates the proportion of surviving patients. p=0.004

**Figure 2:**
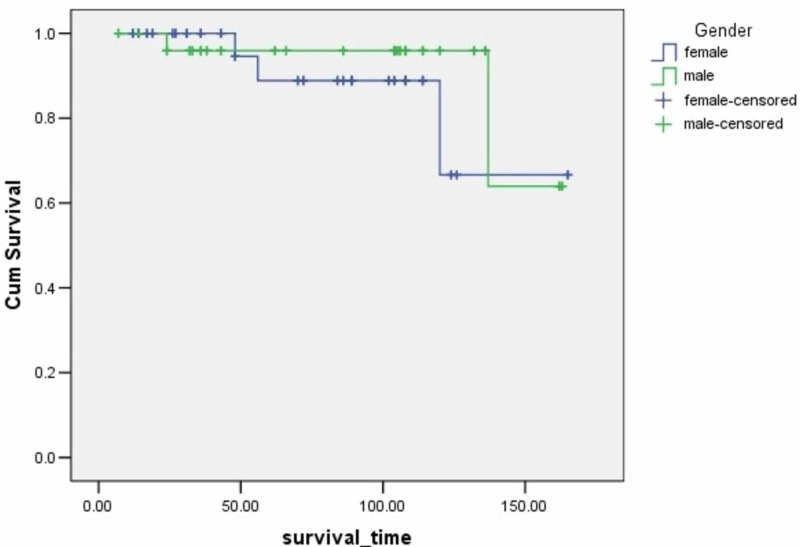
Survival rates based on gender The x-axis indicates months from surgery and the y-axis indicates the proportion of surviving patients. p=0.274

**Figure 3:**
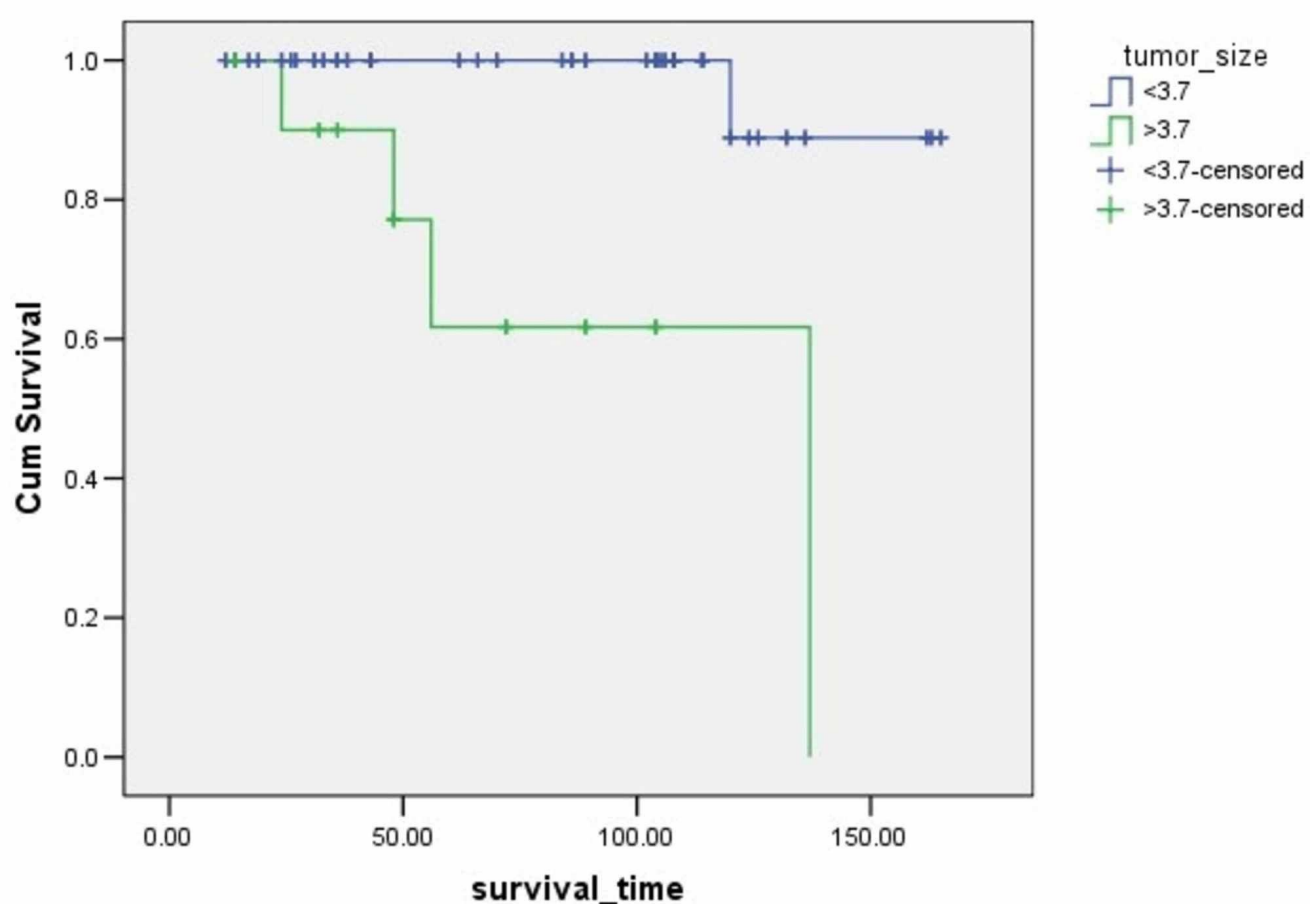
Survival rates based on tumor size The x-axis indicates months from surgery and the y-axis indicates the proportion of surviving patients. p=0.0001

**Figure 4:**
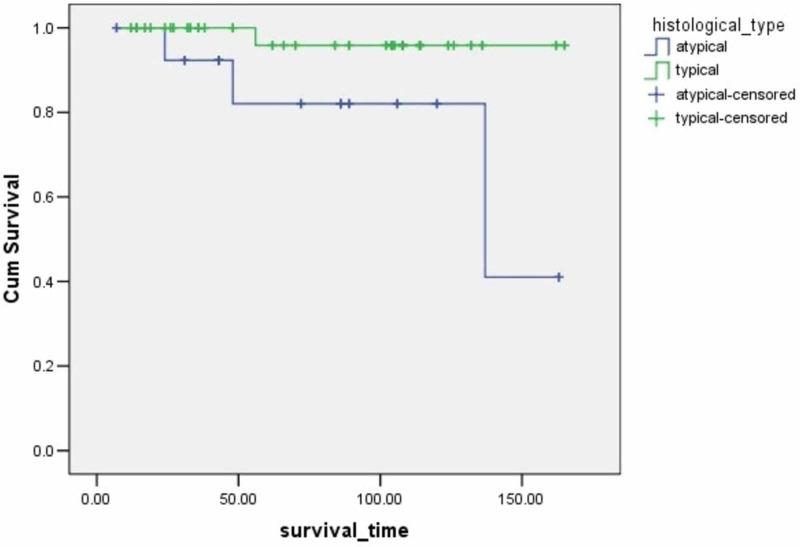
Survival rates based on histological type The x-axis indicates months from surgery and the y-axis indicates the proportion of surviving patients. p=0.006

**Figure 5:**
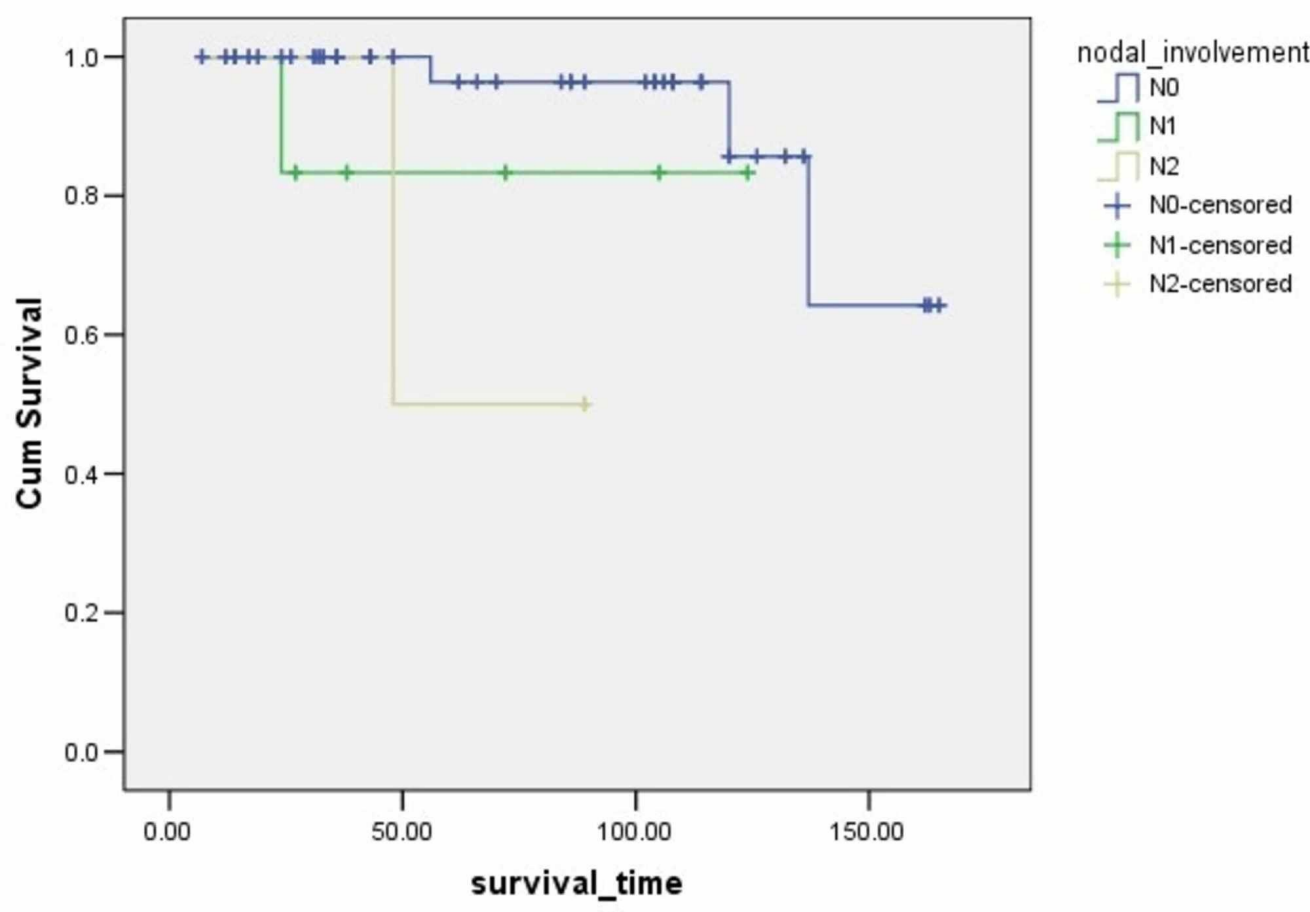
Survival rates based on nodal involvement The x-axis indicates months from surgery and the y-axis indicates the proportion of surviving patients. p=0.001

**Figure 6:**
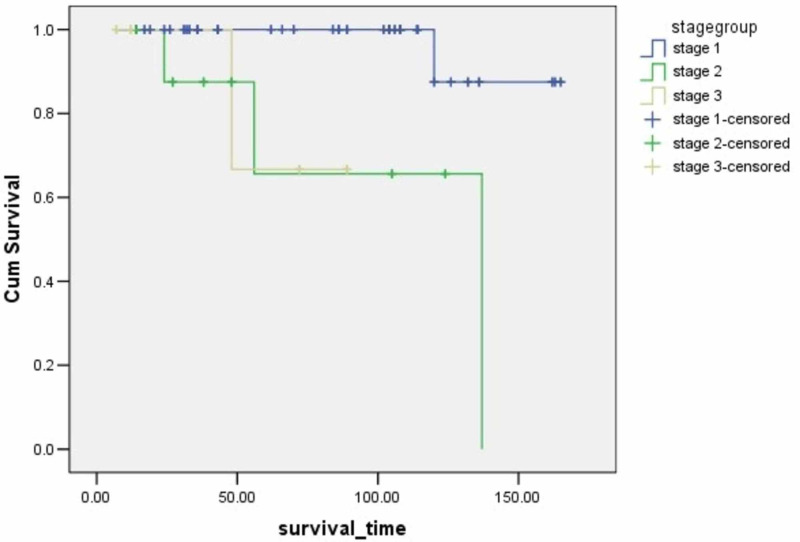
Survival rates based on tumor stage The x-axis indicates months from surgery and the y-axis indicates the proportion of surviving patients. p=0.0001

**Figure 7:**
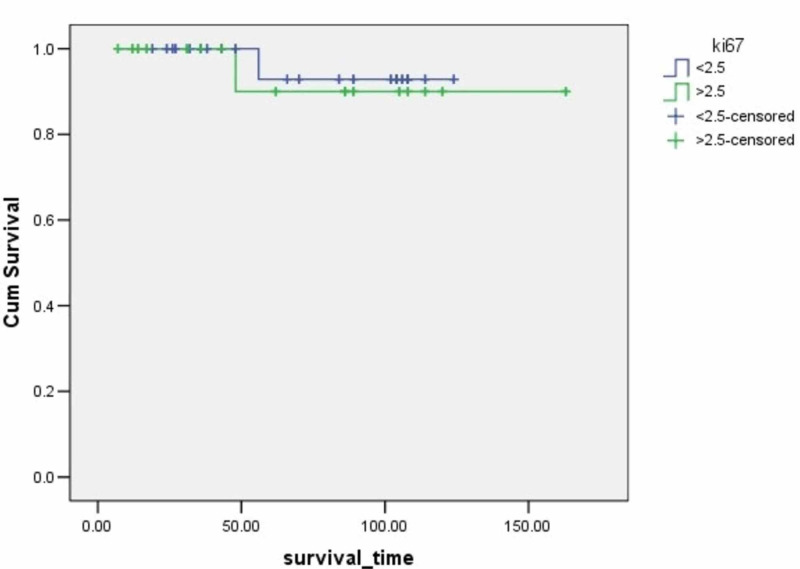
Survival rates based on Ki-67 expression The x-axis indicates months from surgery and the y-axis indicates the proportion of surviving patients. p=0.666

The patients' follow-up ended in March 2019, with a mean time of observation of seven years (96 months). No patients were lost to follow-up. For the follow-up, we performed a history and physical examination and chest/abdomen CT every six months for the first two years and then annually. Of the 108 patients, eight died, all with disease-related death. Recurrence of the same type of tumor occurred, with distant metastases, and they received platinum-based chemotherapy. Two patients had typical carcinoid and six had an atypical carcinoid. Two patients had Stage I disease, four patients had Stage II, and two patients had Stage III, therefore, we cannot say that an advanced stage has more recurrence. The mean survival time for the patients who died from the disease was 77±46.04 months. The survival rates based on age, gender, tumor size, type of carcinoid, stage, nodal involvement, and Ki-67 expression are shown in Table 2 and Figures 1-7.

**Table 2:** Kaplan Meier survival analysis

Variable	p-value	Log Rank	Mean Survival Time (months)	95% CI	Survival Rate (%)
Age (years)	0.004	8.122			
<45			87.4	71.2-103.6	100
>45			66.4	56.3-76.6	90
Ki67 index	0.666	0.186			
<2.5			119.14	112.6-125.6	96
≥2.5			151.5	136.4-166.6	94.1
Histological Type	0.006	7.48			
Atypical			129.8	109.0-150.7	78.6
Typical			160.5	154.3-166.6	97.5
Gender	0.274	1.197			
Male			149	137.1-160.9	92.3
Female			142.4	126.5-158.3	89.7
Tumor Size (cm)	0.0001	27.715			
≤3.7			160	153.5-166.5	97.7
>3.7			101.8	77.5-125.9	63.6
Stage	0.0001	17.6			
I			159.4	152.1-166.7	97.6
II			105.1	76.6-133.7	66.7
III			75.3	59.9-90.8	80
Nodal Involvement	0.001	14.57			
N0			150.2	140.0-160.4	93.3
N1			107.3	86.2-128.4	83.3
N2			68.5	48.4-88.5	50

According to the Cox regression univariate analysis, four factors were correlated to shorter survival: atypical histology, tumor size >3.7 cm, nodal involvement, and advanced stage (Table 3).

**Table 3:** Univariate Cox regression analysis Exp(B): the relative risk between the groups

Variable	Exp(B)	p-value	95%CI for Exp(B)
Age at operation (years) (>45 vs. <45)	0.01	0.178	0.000-8.294
Ki67 index (≥2.5 vs. <2.5)	0.004	0.164	0.000-9.655
Histological type (Typical vs. Atypical)	6.875	0.02	1.357-34.827
Gender (Female vs. Male)	0.498	0.293	0.136-1.823
Tumour size (≤3.7cm vs. >3.7 cm)	0.051	0.0001	0.011-0.243
Stage		0.003	
I (reference) vs II	0.051	0.004	0.007-0.395
I (reference) vs III	0.684	0.117	0.117-4.011
Nodal involvement		0.01	
N0 (reference) vs N1	0.068	0.003	0.011-0.411
N0 (reference) vs N2	0.286	0.232	0.037-2.223

## Discussion

In our study, age was not a factor associated with survival in patients with surgically resected lung carcinoids. Patients' age has been studied as a predictive factor of survival in lung carcinoids in many studies. Filosso PL et al. confirmed that older age is a predictor of worse survival in their study [14]. Age was a variable associated with survival in a study by Ferguson MK et al. [15] and age >60 years old had a statistically significant correlation to worse survival in a study by Cao C et al. [16]. In a study by Chen X et al., the prognosis for lung atypical carcinoids patients with older age became much worse than that of patients with younger age (p < 0.01) [17], and in a study by Lim E et al., age was an independent predictor of survival [18].

Gender is an additional parameter related to lung carcinoids survival in some reports. Filosso PL et al. reported the male gender as a strong negative prognostic factor for pulmonary carcinoids [14] while Beasley MB et al. reported that the female gender was a negative predictor of prognosis (p:0.012) [19]. In the Cox regression univariate analysis of our study, gender was not associated with patient survival.

Another variable associated with patient survival in our study was tumor size. Patients with a tumor size of ≤3.7 cm had better survival as compared to those with a tumor size of >3.7 cm (p=0.0001). Chen X et al. found that patients with the size of the tumor 27.5 mm had significantly shorter survival than those with a tumor size of ≤27.5 mm (p=0.018) [17]. Beasley MB et al. revealed that tumor size 3.5 cm or greater was a negative predictor of prognosis in pulmonary carcinoids [19].

One of the most studied parameters related to lung carcinoid prognosis is the type of carcinoid. Filosso PL et al. found that atypical tumor histology is an independent negative prognostic factor [14]. Beasley MB et al. confirmed that - stratified for stage - patients with atypical carcinoids had a significantly worse survival than those with typical carcinoids (p<0.001) [19]. Ferguson MK et al. in their study for long-term outcomes after resection for bronchial carcinoids reported that survival was related to the histological subtype [15]. Cao C et al. found that atypical histology is related to worse prognosis (p=0.0001) [16], and in a study by Maurizi G et al., atypical carcinoid was confirmed as a negative prognostic factor [20]. In our study, patients with typical carcinoids had better survival as compared to those with atypical carcinoids (p=0.020).

According to several studies, stage and lymph node involvement are associated with patients' survival. Filosso PL et al. demonstrated that nodal deposition was a predictor of worse survival [14]. Chen X et al. in their study showed that lymph node metastasis was an independent prognostic factor of worse survival in patients with atypical carcinoids (p <0.05) [17]. In a study by Ramirez RA et al., patients with N0 staging had an excellent five-year survival rate as compared to patients with N1 and N2 involvement and there was a statistically significant relation between tumor stage and survival [21]. In the current study, nodal involvement and stage are correlated to the patients' survival according to the Cox regression univariate analysis.

The Ki-67 protein is a marker for cell proliferation [22]. Ki 67 expression is correlated to survival in patients with lung carcinoids. Ramirez RA et al. reported that patients with Ki-67 values >10% had a significantly worse prognosis than patients with Ki-67 values <10% (p<0.05) [21]. Costes V et al. revealed that Ki67 of a stained nuclear surface of more than 4% had an independent effect on survival [22]. However, our results did not reveal the Ki-67 index as a parameter related to patients' survival.

The study has some limitations. The study is based on a large number of well-characterized patients according to clinical and laboratory findings, as well as reliable follow-up and surviving data, and is one of the largest studies on pulmonary carcinoids in Greece. However, only a limited number of disease-specific deaths was presented. Therefore, a multivariate analysis could not be performed and maybe a larger study is needed for better results. In addition, due to the limited number of deaths, mortality could not be correlated to the multitude of factors like recurrence or chemotherapy provided. 

## Conclusions

In conclusion, we found that histological type, tumor size, nodal involvement, and stage are associated with survival in patients with surgically resected lung carcinoids without distant metastases. Other parameters, such as age at operation, gender, and Ki-67 index, did not have a role in survival in these patients according to the Cox regression univariate analysis. We confirmed that typical and atypical carcinoids have a different prognosis, as well as tumors with a different size, stage, and nodal involvement. Therefore, correct initial histological diagnosis, classification, and tumor staging is important.
